# *Aspergillus parasiticus crzA,* Which Encodes Calcineurin Response Zinc-Finger Protein, Is Required for Aflatoxin Production under Calcium Stress

**DOI:** 10.3390/ijms9102027

**Published:** 2008-10-29

**Authors:** Perng-Kuang Chang

**Affiliations:** Southern Regional Research Center, 1100 Robert E. Lee Boulevard, New Orleans, LA 70124, USA. E-Mail: perngkuang.chang@ars.usda.gov; Tel. +1-504-286-4208; Fax: +1-504-286-4419

**Keywords:** *Aspergillus parasiticus*, aflatoxin biosynthesis, endoplasmic reticulum calcium ATPase, *crzA*, calcium homeostasis

## Abstract

Two morphologically different *Aspergillus parasiticus* strains, one producing aflatoxins, abundant conidia but few sclerotia (BN9) and the other producing *O*-methyl-sterimatocystin (OMST), copious sclerotia but a low number of conidia (RH), were used to assess the role of *crzA* which encodes a putative calcium-signaling pathway regulatory protein. Under standard culture conditions, BN9ΔcrzA mutants conidiated normally but decreased slightly in radial growth, regardless of illumination conditions. RHΔcrzA mutants produced only conidia under light and showed decreased conidiation and delayed sclerotial formation in the dark. Regulation of conidiation of both *A. parasiticus* strains by light was independent of *crzA.* Increased concentrations of lithium, sodium, and potassium impaired conidiation and sclerotial formation of the RHΔcrzA mutants but they did not affect conidiation of the BN9ΔcrzA mutants. Vegetative growth and asexual development of both ΔcrzA mutants were hypersensitive to increased calcium concentrations. Calcium supplementation (10 mM) resulted in 3-fold and 2-fold decreases in the relative expression of the endoplasmic reticulum calcium ATPase 2 gene in the BN9 and RH parental strains, respectively, but changes in both ΔcrzA mutants were less significant. Compared to the parental strains, the ΔcrzA mutants barely produced aflatoxins or OMST after the calcium supplementation. The relative expression levels of aflatoxin biosynthesis genes, *nor1*, *ver1*, and *omtA*, in both ΔcrzA mutants were decreased significantly, but the decreases in the parental strains were at much lower extents. CrzA is required for growth and development and for aflatoxin biosynthesis under calcium stress conditions.

## 1. Introduction

Several species in the genus *Aspergillus,* most notably *A. flavus*, *A. parasiticus* and *A. nomius*, produce the toxic and carcinogenic aflatoxins [1]. *A. parasiticus* are closely related to *A. flavus*, although the *A. parasiticus* populations are not as diverse as the *A. flavus* ones [2]. *A. parasiticus* isolates are mostly aflatoxigenic. In the U.S., *A. parasiticus* isolates that do not produce aflatoxins usually accumulate *O*-methylsterigmatocystin (OMST). It was estimated that OMST-accumulating isolates accounted for about 2.6% of an *A. parasiticus* population in a southwestern Georgia peanut field [3]. The synthesis of aflatoxins, a group of polyketide-derived secondary metabolites, is a complex process and is regulated at many levels [4–6]. Nutritional factors such as carbon and nitrogen sources [7–11], as well as metal ions and trace elements [12–15], also affect aflatoxin production. Calcium, as a secondary messenger, plays a crucial role in regulating a wide range of physiological functions of cells [16]. The absence of calcium in culture media results in decreased aflatoxin production [17], and blocking of the calcium channels by chemicals inhibits aflatoxin production in *A. parasiticus* [18]. Calcium signaling via the binding of calcium to calmodulin is a well-defined mechanism [19, 20] . The activated calcium-calmodulin complex can interact with a multitude of protein targets, including kinases, phosphatases, and other signaling proteins [21]. Calcineurin is one of the target proteins. It is a serine/threonine protein phosphatase also known as phosphatase 2B [22, 23]. The structure and function of calcineurin are highly conserved among fungi, yeast, plants, and humans. In *Saccharomyces cerevisiae*, calcineurin-dependent signaling is central to the regulation of gene expression via the activation of the calcineurin-responsive zinc finger transcription factor, Crz1p [24, 25]. The *crz1* (*crzA*) genes have been isolated from a few yeast and fungi [24–28]. Calcineurin controls Crz1/CrzA activity primarily through regulation of its nuclear localization by protein dephosphorylation under various stress conditions [22, 24, 28]. The primary role of Crz1/CrzA appears to be in tolerance to metal ion stress [22]. Other functions also have been implicated, for example, hyphae of *B. cinerea* Δcrz1 mutants are unable to penetrate intact plant surfaces [27] and an *A. fumigatus* ΔcrzA mutant is incapable of causing disease [29]. Oxidative stress can cause changes in cytosolic and mitochondrial calcium concentration in *A. nidulans* and tobacco seedlings [30, 31]. Oxidative stress resulting from reactive oxygen species has been suggested to trigger aflatoxin biosynthesis since many antioxidants reduce aflatoxin production [32–34]. In this study, the role of *crzA* in the two types of *A. parasiticus* isolates in response to metal ion stress and in aflatoxin biosynthesis was examined. The results suggest that CrzA is an important determinant to calcium tolerance and has a positive role in aflatoxin biosynthesis under calcium stress.

## 2. Results

### 2.1. Disruption of crzA in morphologically different A. parasiticus strains

The putative *crzA* gene homologue in *A. flavus* (AFL2G_09134.2; http://www.broad.mit.edu/annotation/genome/aspergillus_group/MultiHome.html) encodes a predicted C2H2 zinc-finger protein of 773 amino acids. A BLASTP search of the *Aspergillus* Comparative Database at the Broad Institute showed that it had 69% amino acid identity to *A. fumigatus* CrzA [23, 29] and 67% amino acid identify to *A. nidulans* CrzA [35]. The predicted *A. flavus* CrzA also matches conserved hypothetical proteins of *A. clavatus* (ACLA_027670, 69%), *A. terreus* (ATEG_02928.1, 72%), and *A. niger* (gw1_10.73, 75%). *A. parasiticus* gene orthologues commonly share 98–99% nucleotide identity to the *A. flavus* genes. Therefore, the role of *A. parasiticus crzA* was evaluated by a gene disruption vector constructed based on the *A. flavus crzA* sequence in respective *A. parasiticus* Δku70 recipient strains [36, 37].

**Figure 1.**
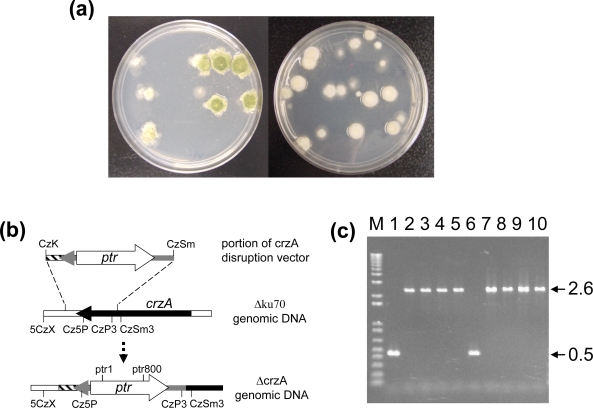
Characterization of *crzA* disruptants. (a) Colony morphology of *crzA* disruptants on regeneration plates. (b) Schematic representation of the expected genomic pattern of a Δ crzA mutant after double-crossover recombination. (c) Genomic patterns of parental strains and ΔcrzA mutants revealed by PCR using primers Cz5P and CzP3. M: DNA 1kb ladder. Lane 1, BN9Δku70; lanes 2–5, BN9ΔcrzA; lane 6, RHΔku70; lanes 7–10, RHΔcrzA.

On pyrithiamine-containing regeneration plates, BN9Δku70 transformants grew normally and produced abundant green conidia, but RHΔku70 transformants barely produced conidia and had a white dense appearance (Figure 1a). Deletion of *crzA* in both strains was confirmed by PCR with primers specific to the *crzA*-coding region, downstream and upstream regions of *ptr*, and regions beyond the expected homologous integration sites. The primer pair ptr1/5CzX, which amplifies a region beyond the left integration site, CzK, and a region of the *ptr* selectable marker (Figure 1b), generated a 1.8-kb fragment from genomic DNA of both types of transformants. Likewise, prt800/CzSm3 generated a 1.9-kb fragment, expected to be present in the right flanking region after homologous integration. These two fragments were not produced from the genomic DNA of each parental strain (data not shown). Cz5P/CzP3 amplified a 0.5-kb fragment from respective parental strains but amplified a 2.6-kb fragment from both transformants (Figure 1c). This indicated that the 2.6-kb portion of the disruption vector which contained the 2.1-kb *ptr* marker had replaced the 0.5-kb region in the resident *crzA* after double-crossover homologous integration. The *crzA* targeting frequency was higher than 95%.

### 2.2. Effects of metal ions, pH and light on vegetative growth and asexual development

**Figure 2.**
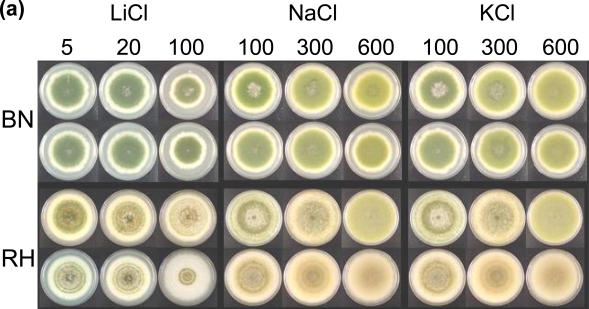

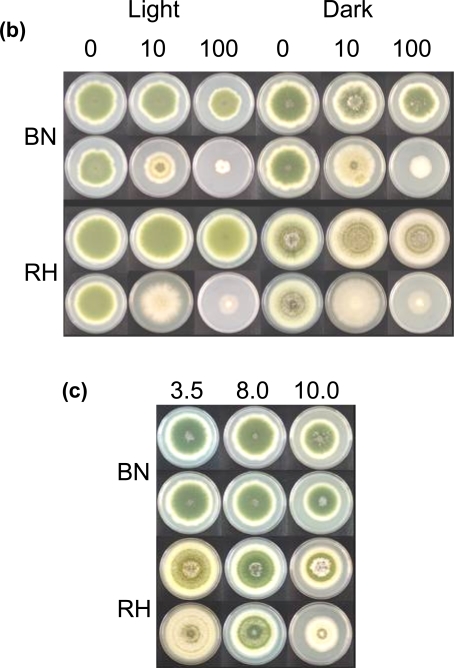
Effects of metal ions and pH on growth and asexual development of ΔcrzA mutants. (a) Tolerance to lithium, sodium and potassium ions at different concentrations (mM) in the dark. (b) Sensitivity to calcium ion at different concentrations (mM). (c) The pH effects in the dark. The upper portion of each panel is the parental strain, and the lower portion is the ΔcrzA mutant.

The *A. parasiticus* strains are sensitive to many metal ions (Figure 2). In the dark at increasing concentrations, addition of lithium, sodium, or potassium to the medium did not affect conidiation of the BN9ΔcrzA mutants, but it decreased conidiation and sclerotial formation of the RHΔcrzA mutants (Figure 2a). Compared to respective parental strains, adding calcium decreased vegetative growth and asexual development of the ΔcrzA mutants regardless of illumination conditions (Figure 2b).

The BN9ΔcrzA mutants and the parental strain had comparable radial growth under acidic and alkaline conditions. For the RHΔcrzA mutants, an inhibitory effect was only observed under pH 10 conditions (Figure 2c). Light had no significant effect on conidiation of the BN9ΔcrzA mutants and the parental strain, but it caused the RHΔcrzA mutants and the parental strain to produce abundant conidia (Figure 2b). The average of the BN9ΔcrzA colony diameters, compared to that of the parental strain, was decreased to 89% (61 ± 2 mm versus 69 ± 0 mm) under light and to 93% (69 ± 1 mm versus 75 ± 0 mm) in the dark. The average of the RHΔcrzA colony diameters, compared to that of the parental strain, was decreased to 93% (70 ± 1 mm versus 75 ± 0 mm) under light and to 96% (79 ± 1 mm versus 82 ± 0 mm) in the dark. The RHΔcrzA mutants in the dark showed a delay in sclerotial development producing many more non-melaninized (white) sclerotia and having less exudates than the parental strain. The RHΔcrzA mutants also produced fewer conidia in the dark estimated to be one third the amount produced by the parental strain (26 ± 8 × 10^4^ versus 82 ± 20 × 10^4^). When examined under a dissecting microscope the RHΔcrzA mutants produced less conidiophores and conidial chains than the parental strain (data not shown).

### 2.3. Calcium supplementation on relative expression levels of calcium-transporting genes

Five genes which encode a sarcoplasmic/endoplasmic reticulum calcium ATPase 2, a calcium-transporting ATPase 1, a plasma membrane calcium-transporting ATPase 2, and two calcium-transporting ATPase 3 were selected for examining how inactivation of *crzA* affected their activities. Of the five genes only the sarcoplasmic/endoplasmic reticulum calcium ATPase 2 gene showed consistent changes in the relative expression levels in both types of the ΔcrzA mutants at 48 h (Table 1).

The relative expression levels of the sarcoplasmic/endoplasmic reticulum calcium ATPase 2 gene in the BN9 and RH parental strains determined from the comparison of gene expression in medium without calcium supplementation to that with calcium supplementation increased about 3-fold and 2-fold, respectively. Under the same growth conditions, the relative expression levels of this gene in the ΔcrzA mutants did not change significantly (less than 50% in both mutants). The relative expression level of the calcium-transporting ATPase 1 gene in the BN9 parental strain increased about 2-fold, but the levels of this gene in the BN9ΔcrzA mutants did not change significantly. The relative expression level of the plasma membrane calcium-transporting ATPase 2 gene in the BN9 parental strain increased slightly, but the levels decreased slightly in the BN9ΔcrzA mutants. The relative expression levels of the ATPase 1 gene in the RH parental strain and the RHΔcrzA mutants decreased to similar extents so did the ATPase 2 gene. The relative expression levels of the two calcium-transporting ATPase 3 genes in the parental strains and the respective ΔcrzA mutants fluctuated widely and could not be correlated with the calcium supplementation (data not shown).

### 2.4. Calcium supplementation on aflatoxin or OMST production and on relative expression levels of aflatoxin biosynthesis genes

**Figure 3.**
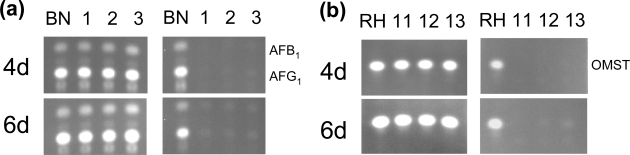
TLC analyses of aflatoxins and OMST produced by parental strains and ΔcrzA mutants. (a) BN9 parental strain and three ΔcrzA mutants. (b) RH parental strain and three ΔcrzA mutants. Left panels are from PDB only, and right panels are from PDB supplemented with 10 mM calcium chloride.

The BN9ΔcrzA mutants produced similar amounts of aflatoxins as the parental strain at day 4 and day 6 in the medium without calcium supplementation (Figure 3a). The RHΔcrzA mutants also produced comparable amounts of OMST as the parental strain in the same medium at day 4 and day 6 (Figure 3b). Calcium supplementation slightly decreased the production of aflatoxins or OMST by respective parental strain, but these metabolites were barely detected from the ΔcrzA mutants during the same time periods.

Quantitative PCR results showed that in general calcium supplementation tends to decrease the relative expression levels of the aflatoxin biosynthesis genes, *nor1*, *ver1*, and *omtA* which represent the early, middle, and late stages of the biosynthesis in both the parental strains and the ΔcrzA mutants (Table 2). Expression of the aflatoxin biosynthesis genes was suppressed in both ΔcrzA mutants compared to the parental strains. At 48 h gene expression levels of the structure genes, *nor1*, *ver1*, and *omtA* in the BN9 parental strain were 5- to 10-fold higher than those of the BN9ΔcrzA mutants. The expression levels in the RH parental strain were 2- to 3-fold higher than those of the ΔcrzA mutants.

## 3. Discussion

*A. parasiticus* is strictly anamorphic. After growth of vegetative mycelia stops, conidiophores start developing upon which asexual conidia are formed. In some *A. parasiticus* strains sclerotia, aggregates of melanized hyphae, are formed, serving as the over-winter structure. The effects of lithium, sodium, and potassium on asexual development of the *A. parasiticus* ΔcrzA mutants were strain-dependent. CrzA was found not necessary for conidiation of the BNΔcrzA mutants under increased conditions, but it is required for conidiation and sclerotial formation of the RHΔcrzA mutants. CrzA apparently is not essential for vegetative growth of both types of *A. parasiticus* because colony sizes of the parental strains and the respective ΔcrzA mutants are comparable even under increased salt conditions (Figure 2). In yeast and other fungi, stress responses of Δcrz1/ΔcrzA mutants to these salts vary considerably. *S. cerevisiae* Δcrz1 mutants are sensitive to lithium, but the *Candida albicans* Δcrz1 mutants are not [24]. In *Torulaspora delbrueckii* loss of Crz1p enhances resistance to lithium but growth is not affected by high sodium concentrations [26]. Loss of CrzA in *A. nidulans* does not affect the vegetative growth, but it affects conidiation at 0.8 M sodium and 0.6 M potassium [35]. At high sodium and potassium concentrations (1.2 M) conidiation and sclerotial formation of a *B. cinerea* Δcrz1 mutant are impaired [27]. The inhibitory effect of the extreme alkaline condition of pH 10 on the radial growth of the ΔcrzA mutants of the two *A. parasiticus* isolates are also clearly different (Figure 2c). The growth of an *A. nidulans* ΔcrzA mutant at pH 8.0 is severely restricted. For *A. fumigatus* inactivation of *crzA* only affects conidiation at acidic and alkaline pH conditions (5.4 and 8.6), but it has no effects on the vegetative growth [28]. These diverse effects of salt and pH stresses on yeast and fungal species and the *A. parasiticus* isolates suggest adaptive responses to specific environmental conditions.

The Glasgow wild-type isolate of *A. nidulans* requires light for conidiation and growth in constant darkness results in only vegetative growth and a lack of asexual conidiation [38]. Nearly all subsequent genetic and molecular studies were carried out using the *veAl* mutant [39] or its derivatives which conidiate in the absence of light. The *veA* gene and red light positively regulates sexual reproduction and negatively regulates asexual conidiation in *A. nidulans* [38]. VeA transport into nucleus is inhibited by white light which suggests that photoreceptors are involved in regulating fungal development [40]. Under light and standard culture conditions (without calcium supplementation), the BN9ΔcrzA and RHΔcrzA mutants conidiate abundantly (Figure 2b) suggesting that light regulates conidiation of *A. parasiticus* and *A. nidulans* similarly and the regulation is independent of *crzA*. In the dark the BN9 parental strain and the BN9ΔcrzA mutants still produce abundant conidia which indicates that *A. parasiticus* VeA functions differently from *A. nidulans* VeA in conidiation. *A. flavus* and *A. parasiticus* strains produce sclerotia in the dark and under continuous red light, but the production is inhibited by continuous white, blue, and green light [41, 42] . Sclerotial development of *A. parasiticus*, which yields a structure considered to be a vestige of sexual cleistothecia that also are woven from specialized hyphae but each contains thousands of ascospores [43], resembles *A. nidulans* sexual reproduction in terms of regulation by red light and VeA [4, 44]. Compared to the RH parental strain the delayed sclerotial formation in the RHΔcrzA mutants suggests that *crzA* probably has a minor role in response to unknown physiological stresses which initiate sclerotial development. Probably, *crzA* may play a role in *A. nidulans* sexual development.

To date, yeast and fungi harboring the Δcrz1/ΔcrzA mutation are hypersensitive to calcium, thereby illustrating the critical role CrzA has in maintaining calcium homeostasis. Endoplasmic reticulum is the cellular compartment for protein posttranslational processing, such as folding, glycosylation, assembly and transport of newly synthesized proteins to the Golgi apparatus. The maintenance of its proper function requires high calcium concentrations. Disturbance of calcium homeostasis in the endoplasmic reticulum is a severe form of stress that interferes with central functions of this structure [45]. Maintaining a high level of calcium storage is also important for calcium signaling, so that in response to certain stimuli calcium can be released into the cytosol. Deregulation of calcium signaling cascades has been shown to result in cell death [46]. Under high calcium concentrations altered expression of calcium transporter genes has been reported for an *A. fumigatus* ΔcrzA mutant [28]. In animals and fungi sarcoplasmic/endoplasmic reticulum calcium-ATPases have been shown to be involved in stress tolerance [47, 48]. This ATPase sequesters and translocates calcium from cytosol to the lumen of the endoplasmic reticulum in replenishing calcium levels thereby playing a central role in maintaining calcium homeostasis. When an exogenous supply of calcium is available, down-regulation of the endoplasmic reticulum calcium-ATPase gene in the parental *A. parasiticus* strains appears to be *crzA*-dependent (Table 1).

Several lines of evidence have suggested that the calcium signaling pathway plays a role in aflatoxin biosynthesis. Rao Praveen and Subramanyam [18, 49] showed that calcium channel blockers and an antagonist of calmodulin causes decreased incorporation of [^14^C]-acetate into aflatoxin B_1_. Jayashree *et al.* [50] reported that during periods of aflatoxin production, protein phosphorylation in *A. parasiticus* is completely lacking but the phosphatase activity of calcineurin is enhanced. This implies that calcineurin-mediated dephosphorylation of regulatory protein(s) or enzyme(s) is associated with aflatoxin biosynthesis. However, the importance of calcium and the mechanism(s) through which calcium has on aflatoxin production remain to be controversial because other studies have found insignificant influences of calcium on aflatoxin production [51, 52] . In this study, calcium added to the growth medium decreased the production of aflatoxins or OMST by the *A. parasiticus* strains. Cercosporin biosynthesis in *Cercospora nicotianaeie* requires the maintenance of endogenous calcium homeostasis and the addition of excess calcium to the medium results in a decrease in cercosporin production [53]. In yeast, Crz1p is the major downstream positive regulator of the calcium/calmodulin/calcineurin-dependent signaling pathway [54]. In response to stresses, dephosphorylated Crz1/CrzA migrates from cytosol to nucleus and activates the expression of genes that contain calcineurin-dependent response elements [28, 35, 54]. Under standard culture conditions *A. parasiticus crzA* is not required for aflatoxin biosynthesis since the ΔcrzA mutants accumulated comparable amounts of aflatoxins or OMST as the parental strains. However, under calcium stress conditions, CrzA has a significant role in maintaining the production of aflatoxins or OMST since the ΔcrzA mutants barely produce aflatoxins or OMST (Figure 3). Compared to the parental strains after calcium supplementation, the relative expression levels of aflatoxin biosynthesis genes, *nor1*, *ver1* and *omtA*, were significantly decreased in the ΔcrzA mutants (Table 2). The mechanism(s) how CrzA, under calcium stress condition, functions to sustain aflatoxin biosynthesis is not clear. Studies have shown that aflatoxin production is positively correlated with asexual reproduction, and *A. parasiticus* mutants that do not produce conidia are unable to synthesize aflatoxins [55, 56]. Conidiation and mycotoxin (aflatoxins, sterigmatocystin) biosynthesis both require inactivation of the G-protein signaling pathway [5]. Cross-talk probably exists between the calcium signaling pathway and the G-protein signaling pathway. Under calcium stress CrzA in the parental strains may down-regulate the expression of some G-protein pathway component genes and initiate asexual development. Constitutive expression of the G-protein pathway related genes due to the loss of *crzA* may favor prolific growth of vegetative mycelia in the ΔcrzA mutants and at the same time suppresses aflatoxin biosynthesis. However, the possibility that the loss of production of aflatoxins or OMST by the ΔcrzA mutants is an indirect effect due to other yet to be identified physiological changes cannot be excluded.

## 4. Experimental Section

### 4.1. Fungal strains

*Aspergillus parasiticus* BN9Δku70 and RHΔku70 were strains conducive to high gene-targeting frequencies [36, 37]. The *ku70* gene, a critical gene of the nonhomologous end-joining pathway, had been deleted in both strains. BN9Δku70 produces aflatoxins and abundant conidia regardless of illumination conditions. RHΔku70 accumulates *O*-methylsterigmatocystin (OMST) as the end product due to a defect in the *ordA* gene necessary for aflatoxin biosynthesis. It produces copious sclerotia and some conidia when grown in the dark.

### 4.2. In silico identification of A. flavus crzA gene

*Saccharomyces cerevisiae* Crz1p amino acid sequence was compared against available fungal ESTs of *Neurospora crassa*, *Magnaporthe grisea*, *A. nidulans*, and *A. oryzae* translated in all reading frames by the tblastn program. Crz1p/CrzA proteins were found to be conserved in the fungi. Genetically *A. oryzae* is closely related to *A. flavus* and known orthologous *A. parasiticus* genes share about 98–99% identity to *A. flavus* genes. A putative *crzA*-containing EST, AoEST06219, was identified from the *A. oryzae* EST database (http://www.nrib.go.jp/ken/EST/db/blast.html), and the corresponding complete *A. oryzae crzA* gene sequence was provided by Keietsu Abe (Tohoku University, Japan), a member of the Japanese *Aspergillus oryzae* Genome Sequence Consortium. *A. flavus crzA* was identified from the later available 5X draft genome sequence of *A. flavus* (http://www.aspergillusflavus.org/genomics/). Restriction analysis of the *A. flavus crzA* gene and flanking regions was carried out using the DNAMAN software (Lynnon Soft, Vandreuil, Quebec, Canada).

### 4.3. Deletion of crzA in A. parasiticus strains

The *crzA* disruption vector was constructed as follows. A 1.4-kb 5’UTR plus *crzA* coding region of *A. parasiticus* were generated by PCR using CzK: CCATTTCATTGCAGGGTACCT and CzSmSp: ATTGCATGCGAGCTGTCGTAGAGTCC. The PCR fragment was cloned into the *Kpn*I and *Sph*I sites of pUC19. The *A. oryzae* pyrithiamine resistance gene (*ptr*), originally amplified from pPTR1 (Takara, Japan) with ptr730P: ATACTGCAGACGGGCAATTGATTACGG and ptr1230P: TTACTGCAGCCGCTCTTGCATCTTTG, was inserted into the *Pst*I site of the resulting vector as a selectable marker. The *crzA* disruption vector was linearized by *Kpn*I and *Sma*I digestion prior to transformation. Transformation and selection on pyrithiamine (PT)-containing regeneration plates were carried out as described previously [36]. Three pairs of primers (a) ptr800: CCTTCTGTGCGAAGCGCTTG and CzSm3: GAGATATACGGCATGTTAGG (b) ptr1: TGGCAGCTGGAGGAGACATG and 5CzX: TGCTGTGTACTAAGTATCTGCC and (c) Cz5P: TTCACCTGGTGCCGACTCCT and CzP3: GCCTGGTGACGAATGATGAG were used to confirm the disruption of *crzA* in BN9Δku70 and RHΔku70.

### 4.4. Determination of colony growth

An aliquot of spore suspension was seeded at the center of each PDA plate (100 × 15 mm). BN9Δku70, RHΔku70, and three ΔcrzA mutants derived from each parental strain were evaluated. Determination of each colony diameter in duplicate plates was performed after growth at 30°C for a week under light and in the dark.

### 4.5. Estimation of conidial production by RH ku70 and the RH crzA mutants

The RHΔcrzA mutants apparently produced decreased amounts of conidia in the dark but not under light. Quantitative analyses were carried out to determine the reduction. To this end, microfuge tubes containing PDA medium (1 mL) were used. An aliquot of each diluted conidial suspension of RHΔku70 and four ΔcrzA mutants was inoculated at the center of two replicate tubes and incubated at 30°C for a week in the dark. One milliliter of 0.01% Triton-X water along with bashing beads (ZYMO RESEARCH, Orange County, CA) was added to each tube. The tubes were agitated by the apparatus of Scientific Industries’ Disruptor Genie™ (ZYMO RESEARCH) to disperse the conidia. Numbers of conidia were determined using a hemacytometer.

### 4.6. Effects of ions and pH on the crzA mutants

Parental Δku70 strains and the ΔcrzA mutants were grown on PDA plates supplemented with various concentrations of lithium, sodium, potassium and calcium (as chloride salts). The pH of PDA plates was adjusted to 3.5 with 10% tartaric acid, or buffered at 8.0 and 10.0 with TRIS at a final concentration of 20 mM prior to sterilization. Cultures were incubated at 30°C in the dark or under white light for a week.

### 4.7. Semi-quantitative thin layer chromatography (TLC) analysis of aflatoxins and OMST

Approximately 10^3^ fresh conidia of each strain were inoculated into PDB (0.25 mL) either with or without 10 mM calcium chloride in a 2 mL microfuge tube and incubated at 30°C in the dark. After 4-d or 6-d growth, methanol (1 mL) was added to each tube to extract metabolites. The tubes were spun for 5 min at the maximum speed, and 0.2 mL of each supernatant was transferred to a new tube. After drying in the air, the extracts were dissolved in acetone (20 μL) and spotted onto BAKER Si250 TLC silica gel plates. The metabolites were resolved with a toluene-ethyl acetate-acetic acid (60:35:5, vol/vol/vol) solvent system and images recorded by a BioRad GelDoc™ documentation system (Bio-Rad, Hercules, CA).

### 4.8. Determination of relative expression levels of plasma membrane (P-type) calcium-transporting ATPase gene homologues in BN9ΔcrzA and RHΔcrzA mutants

A search of the annotated *A. flavus* genome sequence at the Broad Institute (http://www.broad.mit.edu/annotation/genome/aspergillus_group/MultiHome.html) yielded scores of ATPase genes. Five of these genes related to calcium transport were selected and they were AFL2G_02893.2 (sarcoplasmic/endoplasmic reticulum calcium ATPase 2), AFL2G_04426.2 (calcium-transporting ATPase 3), AFL2G_09335.2 (calcium-transporting ATPase 1), AFL2G_10132.2 (calcium-transporting ATPase 3), and AFL2G_10917.2 (plasma membrane calcium-transporting ATPase 2). The expression levels of these genes were determined by real-time RT-PCR in an iCycler iQ5 Multicolor Real Time PCR Detection System (Bio-Rad). Quantitative real-time RT-PCR was performed using SYBR Green I. Stationary cultures were grown for 48 h in PDB with or without 10 mM CaCl_2_. Total RNA was extracted using TRIzol® reagent (Invitrogen, Carlsbad, CA) and treated with DNase I. First stranded cDNA was synthesized with a SuperScript™ III First Strand kit (Invitrogen). The sequences of the 18S ribosomal RNA primers are CATTACCGAGTGTAGGGTTCCTAG and CCGCCGAAGCAACTAAGG. The sequences of the calcium transport gene primers are as follows: 2893, AATCACTACCTGTCTTGCTCTC and CCGTT- GCCAGTCTTGTCC; 4426, GCCTCTCAGCCTTCTCAATC and GCACTCTTCGGACCATTCTC; 9335, AGAATCCGCTGAATCCGATG and GCACCCGTTGAATGAGAGG; 10132, TCTTCGCAA-TCGCAGTGG and AGACAAGCAGGAATCATAGCC; 10917, CGAACTGACTACCGAGAA- GAC and ACCAGCAGCAAGCAAGAC. Amplification conditions included an initial denaturation step at 95 °C for 3 min, followed by 40 cycles, each consisting of denaturation at 95 °C for 10 sec, annealing at 55 °C for 30 sec and extension at 72 °C for 15 sec.

### 4.9. Determination of relative expression levels of aflatoxin biosynthesis genes in the BN9 crzA and RH crzA mutants

Real-time RT-PCR was carried out as described in the above section. The primer sets used were aflR-F: CAACCTGATGACGACTGATATGG, aflR-R: TGCTGCCGCAGCATACC; nor1-F: CCTG- AGGAGACGGTGTATTTGG, nor1-R: CGACCACGGTGCTTTTGG; ver1-F: CGGTGCGCCATTT- TGG, ver1-R: GGTGACCGAACGATACAATTCC; omtA-F: AAGGAGTGGAATTCGCTTATT- ACG, omtA-R: ACCCTTCCTCGCCTTTGC. 18S-F: GCTCTTTTGGGTCTCGTAATTGG, 18S-R: CGCTATTGGAGCTGGAATTACC [57].

## 5. Conclusions

The *A. parasiticus crzA* gene which encodes a zinc-finger protein is dispensable for conidiation under white light and for vegetative growth regardless of illumination conditions. It plays a minor role in tolerance to metal ions of lithium, sodium, and potassium. It is an important component of the calcium signaling pathway. Under calcium stress conditions, the lack of *crzA* in different *A. parasiticus* morphotypes causes severe impairments in vegetative growth and asexual development, and it also decreases aflatoxin production significantly.

## Figures and Tables

**Table 1.** Relative expression levels of three calcium-transporting genes in the parental strains and the ΔcrzA mutants.

Strain	Gene a	Parent	Mutant 1	Mutant 2
BN9	2893	4.4^b^	1.4	1.3
	9335	2.8	1.0	0.9
	10917	1.3	0.8	0.7
RH	2893	2.9	1.2	0.7
	9335	0.4	0.4	0.5
	10917	0.3	0.3	0.3

^a^ 2893 for sarcoplasmic/endoplasmic reticulum calcium ATPase 2 gene, 9335 for calcium-transporting ATPase 1 gene, and 10917 for plasma membrane calcium-transporting ATPase 2.

^b^ Relative expression is determined by the comparison of the normalized transcript level of a strain grown in PDB for 48 h to that grown in PDB containing 10 mM calcium for 48 h. The normalized transcript level from PDB containing calcium is used as a calibrator and is 1.

**Table 2.** Changes of relative expression levels of aflatoxin biosynthesis genes in the parental strains and the ΔcrzA mutants after calcium supplementation.

Strain	Gene	Parent		Mutant 1		Mutant 2	
		48h	72h	48h	72h	48h	72h
BN9	*aflR*	0.78^a^	0.32	0.16	0.06	0.34	0.35
	*nor1*	0.58	0.21	0.05	0.02	0.15	0.10
	*ver1*	0.60	0.17	0.09	0.01	0.13	0.12
	*omtA*	0.53	0.41	0.05	0.01	0.14	0.17
RH	*aflR*	0.76	0.44	0.89	0.15	0.33	0.13
	*nor1*	0.48	0.56	0.24	0.05	0.19	0.06
	*ver1*	0.57	0.99	0.22	0.08	0.14	0.02
	*omtA*	0.41	0.79	0.20	0.07	0.31	0.28

a. The relative gene expression level is determined by the comparison of the transcript level from PDB containing 10 mM calcium to that from PDB only. Data are the averages of two experiments except for the BN9-72h set.
